# Supplementary material for: Are carbon emissions trading and green financial instruments synergistic? -Comprehensive quantitative research based on content analysis
Source: PLoS One. 2024 Mar 7;19(3):e0298601. doi: 10.1371/journal.pone.0298601 (PMC10919601; doi:10.1371/journal.pone.0298601)
Supplement: S1 Data — (ZIP) [file pone.0298601.s001.zip › Data/地级市Z_F工作报告.zip!/╣π╢1⁄2/╜╥╤⌠╩╨/└┤╫╘╣1⁄2╓┌║┼í╛┬φ┐╦╩2╛▌═°í┐_2018.docx]

各位代表：

现在，我代表市人民政府向大会作工作报告，请予审议，并请市政协委员和其他列席人员提出意见。

2017年工作回顾

过去一年，在省委省政府和市委的坚强领导下，在市人大及其常委会和市政协的监督支持下，我们深入贯彻党的十八大、十八届历次全会精神，认真学习贯彻党的十九大精神和习近平新时代中国特色社会主义思想，围绕“五位一体”总体布局和“四个全面”战略布局，坚持新发展理念，坚持稳中求进工作总基调，按照“走正道、出实绩”的要求，以“百日百项大行动”为抓手，积极应对各种困难和挑战，千方百计稳增长、强后劲、惠民生、优环境、谋突破，经济社会发展筑底向好。

——经济运行总体平稳。全年预计（下同）实现生产总值 2155亿元，增长5.3%；规上工业增加值1091亿元，增长2.7%；固定资产投资1642亿元，增长10.5%；社会消费品零售总额1084亿元，增长10.8%；外贸进出口425.2亿元，下降8.7%；地方一般公共预算收入72.8亿元，按可比口径增长0.4%；金融机构各项存款余额2107.05亿元，增长4.5%；各项贷款余额1057.63亿元，增长6.6%。

——重点项目有序推进。26个省级重点项目和209个市级重点项目完成年度投资335.2亿元，中海油粤东LNG项目投产运营并启动管网配套工程建设，揭惠高速一期建成通车，改造国省道38公里，新（改）建地方公路287公里；梅汕客专揭阳段建设进展顺利，潮汕环线、汕湛高速、兴汕高速、揭惠高速市区连接线等工程加快建设；揭阳潮汕国际机场跑道延长及站坪扩建工程开工建设，练江流域综合整治水利工程全面开工；中国石油广东石化炼化一体化项目优化重启，海上风电开发暨研发、总装、运维基地项目签约落户；汕汕高铁揭阳段、大南海公共码头、三江连通工程等项目前期工作加快推进。

——产业转型升级有新成效。实施《“揭阳制造2025”发展规划》，6个省级产业转移工业园和聚集地固定资产投资增长43.6%，新增产业共建项目58个。组织362家规上企业实施技术改造，统筹产业扶持资金1.24亿元，带动全市技改投资361亿元，增长28%。关停和淘汰落后产能及环境污染企业84家，取缔清拆“地条钢”企业22家。实施创新驱动发展战略，新增高新技术企业37家，累计达到102家；新增省级重点实验室1家、省级工程技术研究中心12家、省级现代农业科技创新中心（基地）5家；新增国家知识产权优势企业4家；获认定国家级科技企业孵化器培育单位1家、省众创空间试点单位2家。实施商事登记制度改革，开展经济户口清理和“个转企、企升规、规转股、股上市”企业主体培育工作，新增注册企业7037家，新增规上工业企业64家、规上（限上）商业和服务业企业51家，新增“新三板”挂牌企业2家。着力培育新技术新产业新业态新模式，建设粤东云数据中心，联合腾讯共建示范性智慧城市；大力发展电子商务、快递物流等新业态，快递业务总量跃居全国第15位；新增国家3A级旅游景区和省级旅游度假区各1家。

——城市建设管理有新面貌。省政府批准并印发实施《揭阳滨海新区发展总体规划（2017-2030年）》，编制完成《揭阳城市总体规划（2011-2035年）》，揭阳成为粤东城市群发展和海洋经济带发展的重要中心城市。各县（市、区）加大城市道路建设和公共服务配套建设，市区望江北路西、空港大道等一批市政道路建成投入使用，市儿童公园、黄岐山山门公园等公园建成开放，临江北路西等市政道路项目开工建设。着力加强城市管理，以“创文”为抓手，大力开展打击“两违”、城市清洁、交通整治、绿化美化等行动，成功创建“广东省文明城市”，揭西县获评“广东省县级文明城市”。

——“三农”工作有新提升。农村土地承包经营权确权登记颁证基本完成。完成12.1万亩高标准农田建设和省年度粮食考评任务。“一镇一品”工程取得成效，揭东区埔田镇被评为全国特色小镇，获认定省首批3A级农业公园2个。全面推广农贸市场食用农产品快速检测，化肥农药使用量实现零增长，揭西县被列为国家农产品质量安全县试点。加大农村人居环境综合整治力度，划定禁养区并全面清理禽畜养殖，实施118个乡村绿化美化工程，村庄保洁实现全覆盖，农村生活垃圾有效处理率达94.2%。精准扶贫、精准脱贫扎实推进，162个省定贫困村创建社会主义新农村示范村工作全面铺开，累计实现脱贫8.28万人。全市农民人均可支配收入增长6.3%。

——开放合作有新进展。成功举办第三届中德（欧）中小企业合作交流会、第六期德国中小企业经理人来华交流班、首届中奥企业合作洽谈会，组织对德（欧）合作“百人团”加强交流合作，打造广东对德合作重要平台。42个中德合作落地项目已有9个建成投产，15个正在加紧建设，推进中德金属生态城改革发展，充分发挥电镀园“零排放”技术优势，促进产业转型和高端发展。揭阳港惠来神泉作业区一类口岸通过验收并获准对外开放，国际贸易“单一窗口”上线启用。成功承办广东省第七届粤东侨博会，密切与海外潮团联系，与美国安大略市结为友好城市。

——环境保护力度持续加大。坚决落实中央和省环保督察、国家海洋督察问题整改。保持环保执法高压态势，立案查处案件545宗，关停环境污染企业62家，刑拘409人。全面推行“河长制”，落实流域污染整治责任，加大河涌截污和整治力度。加快污水处理设施和管网建设，9个PPP污水处理厂已有5个通水试运行、4个正在抓紧建设，建成小型污水处理设施2个。市区及普宁2家垃圾焚烧发电厂加快建设。榕江、练江流域考核断面水质有所改善。全年空气优良达标率为94.2%，空气质量六项指标全面达标。绿化揭阳大行动继续推进，新增森林碳汇5万亩，新增水源林1.6万亩，新增森林公园10个，新建生态景观林带13公里，完成“三江”江畔绿化107公里。

——社会民生不断改善。教育创先工作有序推进，增加幼儿园74所，实现普通高中残疾学生、建档立卡等家庭经济困难学生和中职学校符合条件学生免费教育，揭东区实现教育强镇复评验收全覆盖并率先通过省推进教育现代化先进县的督导验收。卫生创强工作全面铺开，市人民医院、中医院、妇幼保健院等扩建工程进展顺利，11家县级公立医院升级建设、5家中心卫生院升级改造项目、38家乡镇卫生院标准化建设和329家公建民营卫生站全部开工。医药卫生体制改革稳步推进，普宁市被定为省医联体建设试点县。成功举办第七届市运会暨第四届学运会，新建体育场地设施示范点77个、省级乡镇文体广场示范点21个。城乡居民养老保险和城乡居民医疗保险实现全覆盖，城乡居民基础养老金提高至120元/人，城乡低保补差、特困人员救助供养、医疗救助住院补助、孤儿生活保障、残疾人“两项补贴”等均达到或超过省定标准，居家养老机构覆盖率提升到69%。棚户区改造项目加快推进，新完成公租房分配2500户。全力协助处置“7 ·6”重大事故，妥善处理“7 ·11”较大事故，安全生产和消防安全形势总体稳定。十九大维稳安保、“飓风2017”“众剑行动”深入开展，盗抢、黑恶、毒品、环境污染等突出犯罪得到有效打击。此外，统计、气象、地震、文化、档案、史志、新闻出版、广播电视、妇女儿童、人民防空、国防动员、民兵预备役、双拥、民族宗教、红十字会、社科、文联等工作也取得了新进步。

——政府建设得到加强。深入学习习近平治国理政新理念新思想新战略，切实增强“四个意识”。自觉接受市人大、市政协及各界监督，定期向市人大及其常委会报告工作，向市政协通报情况，人大代表建议和政协委员提案办结率及满意率均达到100%。全面推进法治政府建设，坚持重大事项集体决策，建立健全政府法律顾问制度，清理并公布已废止和已失效市政府规范性文件99件，办理行政复议案件123件，执法监督约谈干部12名。深化“放管服”改革，取消市级行政许可及中介服务事项35项，调整行政职权57项，全面推行“一门式一网式”政府服务模式，设立24小时自助政务服务区，“智慧揭阳APP”上线服务。12345市民服务热线受理群众咨询、投诉、举报电话12万件。食盐监管体制、城市执法体制改革稳步推进，审计监督和督查问责力度加大，政风行风持续改善。

这些成绩的取得，是省委省政府和市委坚强领导的结果，是市人大、市政协和社会各界监督支持的结果，是全市上下团结拼搏、攻坚克难的结果。我谨代表市政府向全市干部群众、企事业单位、驻军官兵、各民主党派和人民团体，向关心支持揭阳发展的港澳台同胞、海外侨胞、国际友人和各界朋友表示衷心感谢并致以崇高敬意！

在看到成绩的同时，我们也清醒地看到经济社会发展还存在不少短板和弱项：一是经济下行压力持续加大，转型阵痛加剧显现，特别是工业经济、外贸出口、财税收入增速明显回落；二是工业园区集聚度不高，大项目、好项目少，新投产企业不多，存量动能不足，增量规模小；三是城乡规划和城市建设管理水平有待提高，城市配套服务不完善，尚未形成吸引人口、集聚要素、带动发展的城市功能；四是生态环保欠账较多，环境治理任重道远，污水处理设施、垃圾处理设施、污水管网严重不足，河涌治理、农村生态环境整治需要攻坚克难；五是发展不平衡不充分问题突出，教育、卫生、文化、体育等民生领域供给不足，整体脱贫、乡村振兴任务艰巨；六是政府体制机制有待理顺，一些重点领域工作不成体系，资源碎片化、分散化，未能形成统筹发展合力。针对这些困难和问题，我们将认真研究，并采取切实有效措施逐步加以解决。

2018年工作安排

2018年是贯彻党的十九大精神的开局之年和完成“十三五”规划的承上启下之年，做好今年各项工作意义重大。我们要立足揭阳所处发展阶段和揭阳人民对未来美好的期盼，以只争朝夕、时不我待的精神，以抓铁有痕、踏石留印的作风，真抓实干，攻坚克难，推动党的十九大精神在揭阳落地生根、开花结果。

今年政府工作的总体要求是：深入学习贯彻党的十九大精神，以习近平新时代中国特色社会主义思想为指引，按照中央经济工作会议、省委十二届三次全会、市委六届三次全会的部署，坚持新发展理念，坚持稳中求进工作总基调，坚持以供给侧结构性改革为主线，贯彻高质量发展的要求，突出抓重点、补短板、强弱项，打赢防范化解重大风险、精准脱贫、污染防治三大攻坚战，统筹推进稳增长、促改革、调结构、惠民生、防风险各项工作，落实“一四四”工作思路，走正道、出实绩，深入开展“百日百项大行动”，真抓实干，努力把揭阳打造成为粤东新的发展极。

今年经济社会发展的主要预期目标是：地区生产总值增长7.5%以上；规上工业增加值增长7.5%；固定资产投资增长12%；外贸进出口总额增长5%；实际利用外资增长12%；社会消费品零售总额增长12%；地方一般公共预算收入增长8.5%以上；城乡居民人均可支配收入增长8.5%；居民消费价格涨幅控制在3%以内；城镇登记失业率控制在3.5%以内；节能减排约束性指标完成省下达年度计划。

实现上述目标，必须着重在八个方面下功夫：

（一）在发展实体经济上下功夫

切实做好企业服务。把服务企业落地建设、增资扩产、增创效益、做强做大作为第一要务，认真倾听和掌握企业存在的实际困难和发展要求，制定服务企业、促进发展工作方案，系统解决企业落地难、建设难、办证难、人才难、转型难等突出问题。建立“政府领导+职能部门”挂钩服务重点企业制度，市县镇三级分别优选出30家“梁柱”企业，实行本级1位政府领导和1个职能部门共同结对帮扶1家企业，一企一策制定帮扶措施。着力促进中小企业健康发展，推进实施“个转企、企升规、规转股、股上市”工程，梳理建立“企业库”“项目库”“税源库”，分类管理，精准施策，推动企业上规模、出效益。

加强产业平台建设。实施梧桐计划，强化政府主导园区综合开发能力，明确园区管委会主体责任，创新园区开发建设机制，贯彻“统一规划、统一征收、统一建设、统一管理”的要求，市级重点抓好大南海石化工业区、揭阳产业转移工业园、空港经济区临空产业园、揭东经济开发区、中德金属生态城、榕城仙梅民科园等园区建设，提升园区承载能力和服务水平。各县（市、区）也要高度重视产业园区载体建设，推动企业入园发展，制定企业入园和投入产出标准，突出质量效益要求。强化“龙头”带动，依托大项目、大企业开展产业链、价值链招商，推动已投产重大企业服务性产业链条的延伸和挖潜工作。

推进招商引资和产业培育。全力推动中国石油广东石化炼化一体化项目重启建设，大力引进石油化工、新材料、装备制造等产业链项目。推进海上风电开发暨研发、总装、运维基地尽快落地建设，大力发展新能源、海工装备等战略性新兴产业。空港经济区要加快引进基地公司、临空制造、临空物流、基地服务等航空产业项目，积极发展研发设计、现代金融、信息服务等临空服务业。揭东开发区与中德金属生态城要联动发展，推动产业带建设，着力引进环保装备、智能制造等先进制造业。揭阳产业园要加强产业集聚，加快转型升级，促进科技产业集聚发展。榕城仙梅民科园要进一步适应主城区发展需要，提质增效，在促进产业集聚和转型升级的同时，落实环保管理、效益管理。加大产业集聚力度，在产业垂直分工和水平分工中建设产业集群。制定分解下达招商引资计划，以“清单化”明确各地引进企业数量和投资额，每月听取汇报，每季组织检查，年底统一考核，全市力争每季度100个以上项目开工建设。

实施创新驱动和产业转型升级。深入实施创新驱动战略，推动省级高新区扩区更名，申报创建国家高新区。实施高新技术企业倍增计划，力争高新技术企业数量在现有基础上3年内翻一番，建设产业技术转移中心，做实做优科技孵化器、众创空间等“双创”平台，培育服务型科技企业和中小型科技企业。继续推动工业企业研发机构建设，谋划实施新一轮技术改造。加大先进技术和高端人才的引进力度，发挥人才驿站作用，强化产学研合作，落实奖补政策。推动互联网、大数据、人工智能和实体经济深度融合。加快建设粤东云数据中心，申报创建下一代互联网试点城市。加强知识产权保护，加大品牌培育力度。推进质量强市建设，建设省级出口竹笋质量安全示范区，加快建成电线电缆和塑料工艺鞋2个省级质检站。

拓展产业合作发展空间。继续办好第四届中德（欧）中小企业合作交流会，建设驻德中国中小企业中心，抓好中德合作项目落地建设。深度参与“一带一路”建设，积极拓展与俄罗斯、东盟等新兴市场国家的经贸合作。积极推进与粤港澳大湾区、海西经济区等地的产业合作。依托侨乡优势，走出去、引进来，组织到北京、上海、重庆、广州、深圳和港澳地区召开外出乡贤座谈会，做好乡贤回归工作。复制推广自贸区改革创新经验，实施更便利的投资贸易政策，积极发展“互联网+一般贸易”等贸易新业态新模式，实施外贸企业“十百千”提质增效计划，提升对外贸易和利用外资水平。

（二）在提升城市功能上下功夫

以规划设计引领城市发展。切实做好城市规划建设管理的基础性、技术性工作，搭建信息化审批平台，严肃实施城市总体规划，抓紧编制、审批和稳定各层次城乡规划，完善控规全覆盖，推进多规合一，形成全市“一张图”。实施城市多中心发展战略，突出功能区定位，推动市区、城区统一规划、成片开发、聚焦发展。加紧出台加强城市规划、土地管理和城市管理实施办法，从源头上建章立制，规范管理。严厉打违拆违控违管违，全面清理处置问题楼盘，集中清理已供未建各类用地，加强城市土地收储和整备力度，盘活城市资源。编制年度土地储备和出让计划，以清单化、台帐式加强土地开发利用管理，促进集约高效利用。

以项目建设带动城市开发。中心城区要向核心区域聚焦发展，统筹公共配套布局建设，加强基础设施融合对接，推动中心城区成片成体系成规模。加大城市道路投资建设力度，以市区“八路一河一桥”为重点，推进建设莲花大桥及其连接线，开工建设进贤门大桥、新城大桥和机场连接线、京灶大桥及其连接线，推动榕城区按照统一标准与空港经济区共建望江北路、临江南路和进贤门大道，促进市区东部片区成片开发、北部片区和中部片区一体发展。聚焦榕江新城开发建设，加快环岛路和景观带、城市道路与公共配套设施建设，加快重点组团项目的土地储备出让和开工建设。加强与招商局集团沟通对接，稳定滨海新区新城起步区和产业起步区规划，健全开发建设体制机制，争取一批先行性市政项目和产业项目落地开工。

以功能配套提升城市品质。加快完善城市教育、医院、文化体育设施、公园、绿地、公厕等公共配套，抓好城区电网、路网、管网、光网改造升级，确保市政生活配套与新区建设和旧城更新同步推进。推进城市老旧社区和农村社区“微改造”，实施棚户区改造和低效用地二次开发，提升城市开发建设水平。引导传统专业市场、物流园区调整优化布局，加快市区“退二进三”，积极发展现代服务业，引进建设城市综合体、会展中心、酒店、高端商务区、优质居住小区等配套设施，增强城市产业发展能力，满足市民生活消费需要。

以机制创新强化城市管理。理顺城市管理体制，落实权责下沉。大力开展国家卫生城市、国家森林城市、国家生态文明建设示范市、全国综治（平安建设）优秀市、全国文明城市“五城同创”活动，联合腾讯共建示范性智慧城市，推动城市治理现代化、信息化，让交通严起来、露土绿起来、绿化拦起来、灯光亮起来、停车管起来，确保每一寸土地、每一件事都有人管，让城市生活更美好。

（三）在交通建设管理上下功夫

巩固交通枢纽地位。推进揭阳潮汕国际机场跑道及站坪改造升级工程，筹划扩建机场航站楼，建设综合交通换乘中心，争取开辟新航线，提升机场服务能力。进一步理顺沿海沿江港口资源和各类码头建设计划，合理安排港口和码头功能，加强揭阳港神泉港区口岸核心能力建设，发展通江达海水路运输。加快梅汕客专揭阳段建设，启动广梅汕铁路改线工程，推动“四站合一”和相关配套设施同步规划、同步建设，配合汕尾-汕头铁路揭阳段先行工程开工建设。推动揭惠高速全线及市域连接线通车，抓紧潮汕环线、汕头至湛江、兴宁至汕尾3条高速公路揭阳段及其连接线建设。新建主干配线光缆1200皮长公里以上，基本实现百兆光纤和4G网络行政村全覆盖，构建网上高速公路。

完善路网体系提升建设水平。编制道路交通建设规划和三年行动计划。完成国道539线、省道236线改造工程，开工建设国道206线改线、国道238线和省道335浦东平交至霖磐高速口改建、惠来华湖互通立交至省道235连接线等6个项目，推动有条件的地方公路升级为国省道，力争全年新（改）建地方公路200公里以上。按照市政道路标准规范提升城市道路建设管理水平，推进揭东护堤路、玉都大道、新城大道等市政道路项目建设，梳理连接机场、高铁、高速公路等出入口道路规划建设需要，统一规划，统一标准，统一建设。按照旅游公路标准，切实加强郊区道路和农村公路建设改造，同步规划建设道路两旁绿化带、道路标识系统等。按照“四好”公路的要求，切实把农村公路建好管好护好运营好。

推进交通运输体制改革和加强行业管理。统筹客货运站点布局，推进综合交通枢纽站建设。理顺国有交通运输企业产权体制，重组交通运输主体，加强行业管理，规范运输市场秩序。优化市区公交线路，扩大公共交通城乡覆盖范围，改革公交补助机制，方便群众出行。增加新能源公交车投放，倡导共享交通、绿色出行。编制市区停车场所建设专项规划，增加中心城区公共停车位，鼓励建设经营性停车场，缓解城市停车难题。实施公路安全生命防护工程，优化道路交通安全标识系统，加强轮船渡口安全管理，建设公安交通集成指挥平台，持之以恒推进城市道路交通秩序大整治，严管严治严惩交通违法违规行为。

（四）在县域发展和乡村振兴上下功夫

突出县域建设发展重点。榕城区要重点抓好问题楼盘处置和土地清理整备，统筹资金推进“五路一河”和污水管网建设；落实古城保护和有序推进城市更新，加强城市公共服务供给，大力发展现代服务业，向东与空港经济区实现连片发展。揭东区要围绕融入和服务核心区，强化基础设施和公共服务配套；抓好揭东经济开发区、中德金属生态城两个产业园区建设运营，为市区产业集聚提供支撑。揭阳产业园和空港经济区要突出发挥好经济功能区作用，强化规划引领、开发建设、城市运营管理、产业发展职能，同时按照授权委托的要求，履行好行政区域服务管理职能，加强镇、村社会事业和社会服务管理。普宁市要引导纺织服装业落实环保措施，推进纺织服装产业园建设，以信息化、智能化为重点实施技术改造，促进产业转型升级；大力发展大健康产业，促进产业向价值链高端延伸；加快城乡污水处理设施和截污管网建设，推进练江流域综合整治工程；落实土地清理整备，统筹资金加快新区规划建设；下大力气加强城市管理，同时高度重视抓好消防安全工作。揭西县要以打造全域旅游示范区为目标，加强生态保护，抓好新农村建设和扶贫攻坚工作，建设美丽城乡；整合旅游生态资源，实施富民工程，补齐发展短板。惠来县要以粤东新城为重点，保障新区加快规划建设，推动沿海港口、产业园区、中心城区有机融合，促进海洋经济发展；同时要重典整治涉毒犯罪，以良好发展环境服务大项目落地建设。大南海石化工业区要依托中石油项目，加快园区开发建设，加强石化产业链招商，争取有一批新项目落地建设。普侨区要积极对接普宁市，大南山侨区要对接揭阳滨海新区，实现联动发展。

推进县级产业园区建设。各县（市）要聚焦主导产业，推进产业园区和产业集聚区建设，进一步完善管理体制和运营机制，加快园区扩容提质和基础设施建设，强化产业链价值链招商，推动产业集聚转型升级。普宁市要重点抓好英歌山工业园和纺织服装产业园，惠来县要重点抓好临港工业园，揭西县要重点抓好电线电缆产业园等园区建设。同时要加快专业镇转型升级，积极培育特色小镇，支持每个县（市）试点建设2-3个小型专业产业园区，推动村镇企业成片转移入园。

改善县域城乡人居环境。把城区、镇区规划好、建设好、管理好，提升形象、提高城镇化水平。推动规划进村，划定村庄边界，规范村民住宅建设，加强旧屋修缮、新屋戴帽，规范立面要求。推进城乡人居环境综合整治，基本完成省“三清除、三拆除、三整治”任务。实施城乡内河整治，提升村道硬化覆盖率。完善“户收集、村集中、镇转运、县处理”的垃圾收运处理模式，实现生活垃圾全部消纳处理。加强养殖场环保设施建设，倡导畜禽养殖零排放，推进化肥农药使用量零增长行动。推进农村“厕所革命”，全面消灭农村旱厕，加强农业旅游景区标准化公厕建设。动员乡贤和华侨力量，完善乡村公共设施，加强乡村治理，完善民主监督机制，抓好移风易俗，促进乡风文明。

激发乡村振兴内生动力。按照“一县一业”“一镇一品”的思路，打造揭西茶庄经济、揭东竹笋经济、普宁花木经济、惠来海鲜经济等优势特色产业，培育农村主导产业，大力引进培育现代农业经营主体，推动农业种养加工、旅游体验等综合开发。加快建设神泉示范性渔港，规划建设广垦卅岭生态农业公园，打造一二三产业融合示范基地。发展“互联网+农业”，推动农产品上网。加强农民技能培训和农村青年创业培训，促进农村劳动力转移就业和农民增收。深化农村改革，划定粮食生产功能区和重要农产品生产保护区，做好农村土地承包经营权确权登记颁证后续工作，启动农村集体产权制度改革试点，为农村经济适度规模发展和特色发展注入活力。

全面决胜脱贫攻坚。建立县（市、区）负主体责任，市统筹解决重点难点问题，对口帮扶为辅助的扶贫工作机制。加强精准识别，完善扶贫建档立卡，统筹抓好本地就业、转移就业、自主创业和扶贫产业，按时按质完成三年脱贫攻坚任务。完善农村保障体系，积极防控因贫辍学、因病返贫，落实扶贫政策“兜底”，重点关注五保、低保、残疾人等弱势群体，推进农村危房改造。结合乡村振兴计划，以扶贫攻坚为龙头，发挥农、林、水、渔力量，统筹好对口帮扶和乡贤资源，加大财政投入力度，推动162条省定贫困村创建社会主义新农村示范村。

（五）在加强生态环保上下功夫

2018年是环境保护决战决胜年，要坚决落实中央和省环保督察、国家海洋督察问题整改，理顺工作机制，压实工作责任，综合施策，切实提高水、大气、土壤防治成效，努力建设美丽揭阳。

加大环境污染治理。落实水、大气、土壤污染整治实施方案和行动计划，明确整治目标，建立清单台帐，以考核和问责倒逼工作落实。全面推行“河长制”，明确职责，按照问题在水里、根源在岸上的思路查清污染源，加强监测，强力整治工业污染、农业面源污染、生活污水污染，完成市区5条黑臭水体整治，努力实现水质达标。加快黄标车淘汰进度，严管建筑工地扬尘、农村垃圾、秸秆焚烧、泥头车运输撒漏等行为，确保空气达标。开展土壤质量调查，清理农田沟渠，严防土地污染。雷厉风行、铁腕整治，从快从严从重打击环境污染行为。开展污染源入户普查，建立环保大数据，做好排污费改税工作。

加快环保设施建设。开展污水处理设施及管网建设大会战，制定城市污水处理设施和管网建设清单，加快污水处理设施建成投入使用，配套完善截污管网，提高污水收集能力，力争全年全市新建污水处理管网120公里以上。加强产业集中治污，推动“零排放”电镀园（二期）、纺织印染环保综合处理中心建成运营，推进凉果加工污染集中治理。推进4个整县村镇污水处理设施项目建设，科学精细治污；加快建成市区及普宁2个垃圾焚烧发电厂、市区东径外草地垃圾填埋场扩建工程、市区污泥及餐厨垃圾处置中心等项目。

加强生态保护修复。实施“三江连通”工程，打造韩江、榕江、练江互济大水系，提升水资源综合利用。全面完成榕江大围达标加固工程、引榕灌区改造工程。推进榕江南北河、练江、枫江综合治理和龙江保护，精心组织水岸同治工程，确保水清岸绿路净，展现“五河毓秀”风光。组织实施第三次全国土地调查，严格耕地保护，推进水田垦造和高标准农田建设。开展土地矿产卫片执法检查，从快从严从重打击违法用地、私挖滥采等破坏环境生态行为。编制我市海洋生态保护规划，严守生态红线，加强海岛巡查保护，严禁违法填海，推进自然岸线修复和美丽海湾建设。

实施绿满家园行动。开展“五路绿化”行动，在高速公路、国道、省道、县道和乡村道路两侧，大规模开展沿路植树活动，打造多层次、多品种的道路绿化景观。推进“一河两岸”植树造景和水源涵养林建设。加快夕阳红等9个市区公园建设，推动建设“一镇一公园”“一镇一绿地”，同时规划建设一批社区型小公园、小绿地。开展全民种树，推动森林围城、花木上路、树林入村，推进国有林场改革，加强护林队伍建设，强化森林和自然保护区保护，努力创建国家森林城市。

（六）在保障民生事业上下功夫

把老百姓关心的事情一件一件办好，切实提高保障和改善民生水平。

推进教育创先工作。推进城乡义务教育一体化改革发展，动态研究学校建设布局，优先抓好新城区配套学校建设，分类妥善处理好乡村麻雀学校，确保学校跟着城市规划和人口走。统筹全市高中阶段学校扩容提质，加快高水平高质量普及高中阶段教育。推进学前教育三年行动计划，提升公办幼儿园和普惠性民办幼儿园覆盖率，切实规范办园行为。加快职业教育发展，规划创建一所应用型本科大学，支持揭阳职业技术学院创建高水平职业院校，推动市高级技工学校创建技师学院，支持揭阳卫生学校提升办学层次。深入推进“强师工程”，培育名教师名校长。实施中小学校长公开竞聘和中小学教师“县管校聘”管理改革，促进教育资源合理流动。建立健全学生资助制度，扩大教育扶贫补助对象，完善保学控辍体系。贯彻民办教育新法新政，支持和规范社会力量办学。

完善医疗卫生服务。加快市人民医院、中医院、妇幼保健院、疾控中心等市级医疗卫生项目扩建工程，推进11家县级公立医院升级建设和5家中心镇卫生院升级改造项目，完善各级医疗卫生服务体系。继续推进公立医院综合改革，加快建设以三级医院为龙头、所有公立医院全部参与的医联体，建立健全分级诊疗制度。推进家庭医生签约服务，实施国家公共卫生项目，加强重大传染病防控。坚持中西医并重，传承发展中医药事业。加强医德医风建设，培育壮大全科医生队伍，建立健全医疗人员引进培育和流动机制。支持和规范社会力量办医，发展康养产业。制定实施《“健康揭阳2030”行动计划》，深入开展爱国卫生运动，倡导健康文明生活方式。

发展文化体育事业。加强公共文化设施建设，理顺市文化中心建设历史遗留问题，分步骤有计划统筹推进市大剧院、博物馆、群众艺术馆、岭东美术馆、青少年活动中心和综合服务楼建设安排，完善配套功能，把市文化中心建设成为文化艺术教育交流示范基地。加强文物保护利用和文化遗产保护传承，加快古城保护开发利用，修缮丁氏光禄公祠，支持“非遗”传承保护基地建设，推动潮汕特色文化创新发展。持续组织举办丰富多彩、喜闻乐见的群众性文体活动，活跃群众文化生活。加强史志档案工作，推进市数字档案馆和县级综合档案馆达标建设。申办2021年省第五届体育大会，规划建设市体育中心，开工建设市体育运动学校，推动体育进社区、进乡村，发展群众体育，推动全民健身。

支持就业创业。落实就业创业补助政策，广泛开展职业技能培训，着力解决结构性就业矛盾。创新公共就业服务，推动高校毕业生多渠道就业创业，促进农村劳动力有序转移。落实退役士兵安置和权益保障。完善政府、工会、企业共同参与的协商协调机制，构建和谐劳动关系。大力弘扬企业家精神、劳模精神、工匠精神，鼓励创业带动就业。

完善社会保障体系。实施全民参保计划，推进企业养老保险省级统筹和扩面征缴，力争城镇职工养老保险参保率达到65%、缴费率达到60%以上。完善城乡居民基本医疗保险和大病保险、大病救助制度，推动实现城乡居民基本医疗保险市级统筹。强化民生“兜底”，完善社会救助体系。启动引龙工程，推动市区供水一体化，推进村村通自来水工程。

办好民生十件实事。一是完成市区“八路一河一桥”项目建设。二是创办1所市直示范性公办幼儿园，公办幼儿园和普惠性民办幼儿园覆盖率提高至68%。三是完成38家乡镇卫生院标准化和300家村卫生站建设，人均基本公共卫生服务经费提高至50元以上。四是新建20个以上乡镇文体广场示范点、30个以上体育场地设施示范点、300个基层综合性文化服务中心。五是新建10个残疾人社区康园中心，实施2000户贫困重度残疾人家庭无障碍改造。六是新增市区公共免费停车位1000个，在全市1100个路口和100所学校门前完善交通标识。七是推动每个乡镇（街道）至少建设1个公园，每个行政村（居委会）配套1个微型消防站。八是扩建市儿童福利院、市救助管理站，规划建设市救助安置中心。九是改造农村危房380户以上，基本建成城市棚户区安置房600套，完成公租房分配入住900户。十是新建1所老年大学，推动老年教育发展。

同时，做好新闻出版、广播电视、气象、地震、军民融合、国防动员、民兵预备役、双拥、人防、民族宗教、妇女儿童、红十字会、社科等工作。

（七）在打造发展环境上下功夫

营造高效便利的政务环境。深化“放管服”改革，实行审批事项、服务事项、便民事项清单管理，进一步压缩审批项目、审批环节、审批时限，确保该放的权放到位。深化“一门式一网式”政府服务改革，建设网上办事大厅企业专属网页和市民个人网页，全面推行数据共享和并联审批，加快推进“数字政府”建设。推动市县镇村四级政务“窗口”服务在线监控全覆盖，全方位规范政务服务行为，努力让老百姓上门办事“只跑一趟路”。

营造公平有序的市场环境。发挥市场在资源配置中的决定性作用和更好发挥政府作用，既要有效市场，更要有为政府，深化供给侧结构性改革，全面落实降低制造业企业成本等政策措施。组织开展第四次经济普查，运用好经济户口清理成果，完善企业数据综合管理平台，实施“双随机、一公开”制度，规范和减少企业多头接受检查，建设联合征信系统；加强依法治税、数据管税力度，实行国地税业务联办。全面推进公共资源交易信息化。严厉打击制假售假、走私贩私、偷逃骗税、逃废债务等经济犯罪活动，维护市场经济秩序。发挥行业协会功能作用，强化行业自律，引导市场主体诚信经营、公平竞争。

营造和谐稳定的社会环境。深入开展社会治安综合治理“众剑行动”，依法严厉打击黄赌毒黑拐骗、乡匪村霸、“两抢一盗”、电信网络诈骗、网络传销等犯罪，建设社会治安防控信息资源综合管理平台和电子围栏，推进社会治安和城市执法网格化管理，增强市民安全感。深入开展涉毒专项整治，推动全市毒情形势持续好转，确保惠来如期“摘帽”。扎实推进“安全大过天，幸福过大年”专项行动，全面完成既有建筑消防安全综合治理，强化安全生产、消防安全、道路交通安全工作，坚决遏制重特大安全事故。强化食品药品安全监管，完成食品抽检每千人不少于2批次任务，改革生猪屠宰管理体制，抓好家禽“集中屠宰、冷链配送、生鲜上市”工作，推进揭西县国家农产品质量安全县试点建设，加快创建省食品安全示范县。建设市应急指挥中心，提升预防和处置突发性事件能力。创新基层治理模式，压实基层维稳责任，落实信访维稳工作制度，加强基层司法所规范化建设和“一村一法律顾问”工作，推进基层治理法治化，切实营造良好的社会环境。

（八）在加强政府建设上下功夫

组织开展政府系统“不忘初心、牢记使命”主题教育，牢固树立“四个意识”，强化“四个自信”，增强政府工作本领，提升政府施政水平。

全面推进依法行政。自觉接受市人大、政协和社会监督，完善政府与人大代表、政协委员联系沟通机制，做好人大代表建议和政协委员提案办理。出台政府部门权责清单动态管理办法，严格执法行为，规范行政权力运作。推行政府法律顾问制度向基层延伸，实现市、县、镇三级政府法律顾问制度全覆盖。加强行政复议和行政应诉工作，切实履行人民法院涉行政机关生效裁判。

深化改革促发展。强化改革责任担当，推进各领域探索创新，用改革思维和改革办法推动改革落地生效。深化政府机构改革，积极稳妥地推进职能整合，厘清部门职责，建设责任政府。建立政府投资项目代建制，推动政府投资项目统一实施。组建市级行政审批机构，实行审批事项统一管理。深化投融资体制改革，优化财政投入方式和效果，着力放大财政资金的引导效应和产出效益，同时引导社会资本投向补短板、调结构、促创新、惠民生等领域。深化地方金融监管体制改革，强化地方金融监管和风险防控处置。深化国有企业改革，在扎实推进国有“僵尸企业”出清的同时，整合全市国有资产，重构市水务集团和城投公司等，做大做强市属国有企业，更好地发挥国有企业在经济建设中的作用。

着力理顺工作机制。完善各项工作机制，增强政府管理的系统性、整体性和协同性，形成工作合力。市级要进一步发挥好规划引领、宏观统筹、具体指导、监督检查的作用，县（市、区）要履行属地职能，发挥好主体作用。进一步理顺市县（区）镇财权、事权管理关系，编制统筹所有财政资金的分地区、分行业管理预算，建立健全以支出项目和支出科目统筹资金安排的综合预算管理，集中财力保障民生、保障建设发展需要，同时加强财政资金监管和审计监督，切实做到生财有道、理财有方、用财有规。

激发干事创业激情。大力弘扬敢抓敢管敢担当的精神，把雷厉风行和久久为功结合起来，不等不靠，主动作为，真抓实干。切实转变思想观念，主动拥抱新时代，以更加开放的心态推动要素集聚，促进产业和城市发展。切实转变工作方式，系统工作要精心制订实施方案，工作任务要项目化、清单化，重点项目要工程化，建立工作台帐，强化工作措施，一件事情一件事情抓落实。切实转变工作作风，按照严、实、细的要求，以严的标准倒逼工作落实落细。加强政府人力资源管理，提高专业素养，提升业务能力，努力打造一支懂建设、会管理、善经营的干部队伍。

加强政府廉政建设。严格落实中央“八项规定”和国务院“约法三章”，全面贯彻党风廉政建设“两个责任”，持之以恒整治清除“四风”问题，建立“亲”“清”新型政商关系，政府与企业打开前门沟通，摆上台面解决问题，教育广大干部既要勇于担责，又要习惯在监督下工作，学会在约束下生活，以良好政风作风取信于民。

同志们！使命呼唤担当，实干成就梦想。幸福是努力创造的。让我们更加紧密的团结在以习近平同志为核心的党中央周围，雷厉风行，久久为功，撸起袖子加油干，在新时代展现新气象、作出新作为，用实绩实效向全市人民交上一份满意的答卷！
